# Comprehensive Study of the Risk Factors for Medication-Related Osteonecrosis of the Jaw Based on the Japanese Adverse Drug Event Report Database

**DOI:** 10.3390/ph13120467

**Published:** 2020-12-16

**Authors:** Shinya Toriumi, Akinobu Kobayashi, Yoshihiro Uesawa

**Affiliations:** 1 Department of Medical Molecular Informatics, Meiji Pharmaceutical University, Kiyose, Tokyo 204-8588, Japan; 2 Department of Pharmacy, National Hospital Organization Kanagawa Hospital, Hadano, Kanagawa 257-8585, Japan; kobayashi.akinobu.tk@mail.hosp.go.jp

**Keywords:** medication-related osteonecrosis of the jaw (MRONJ), atypical femoral fracture, bisphosphonates, denosumab, spontaneous reporting system, Japanese Adverse Drug Event Report (JADER), pharmacovigilance, logistics regression analysis, principal component analysis, cluster analysis

## Abstract

Medication-related osteonecrosis of the jaw (MRONJ) is associated with many drugs, including bisphosphonates (BPs). BPs are associated with atypical femoral fractures and osteonecrosis of the external auditory canal. Thus, many drugs are reported to cause adverse effects on bone. This study aimed to investigate the effects of drugs and patient backgrounds regarding osteonecrosis-related side effects, including MRONJ. This study used a large voluntary reporting database, namely, the Japanese Adverse Drug Event Report database. First, we searched for risk factors related to MRONJ using volcano plots and logistic regression analysis. Next, we searched for bone-necrosis-related side effects using principal component and cluster analysis. Factors that were significantly associated with MRONJ included eight types of BPs and denosumab, prednisolone, sunitinib, eldecalcitol, raloxifene, letrozole, doxifluridine, exemestane, radium chloride, medroxyprogesterone, female, elderly, and short stature. Furthermore, antiresorptive agents (i.e., BPs and denosumab) tended to induce MRONJ and atypical femoral fractures by affecting osteoclasts. We believe these findings will help medical personnel manage the side effects of many medications.

## 1. Introduction

Since the first report by Marx [1], antiresorptive agents (i.e., bisphosphonates (BPs) and denosumab), or antiangiogenic agents (e.g., bevacizumab and tyrosine kinases inhibitors) have been reported to be associated with medication-related osteonecrosis of the jaw (MRONJ) [2,3,4,5]. Patients may be considered to have MRONJ if they present with the following characteristics: (1) current or previous treatment with antiresorptive or antiangiogenic agents; (2) exposed bone or bone that can be probed through an intraoral or extraoral fistula in the maxillofacial region, which has persisted for over 8 weeks; (3) no history of radiation therapy to the jaws or obvious metastatic disease to the jaws [2]. Meanwhile, corticosteroids and novel immunomodulators have also been reported to affect osteonecrosis of the jaw [6,7]. BP is also reported to cause bone-related side effects, such as atypical femoral fractures and osteonecrosis of the external auditory meatus [8,9]. Many other drugs exhibit adverse effects on bone.

MRONJ significantly reduces the quality of life of patients [10,11]. Discontinuation of resorption inhibitors is recommended for patients with risk factors, such as oral surgery and corticosteroid treatment [12]. Conversely, MRONJ is rare, and the benefits of drug treatment clearly outweigh the risks in many cases. Therefore, treatment is recommended when the risks of MRONJ are minimal [13]. If risk factors for MRONJ are clarified, the management of side effects can be improved. This study examined two key clinical questions concerning risk factors associated with MRONJ and the effects of these drugs on other osteonecrosis-related side effects using the Japanese Adverse Drug Event Report database (JADER).

## 2. Results

### 2.1. Presentation of Data

**Figure 1:**
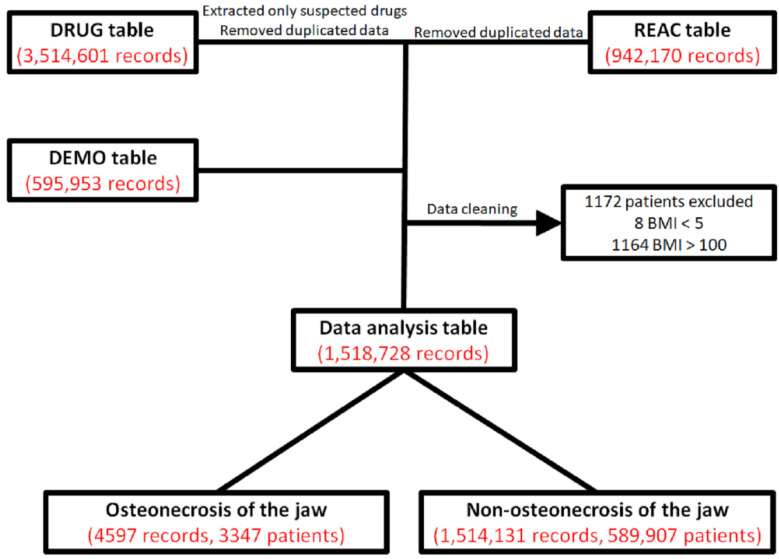
Flowchart for the construction of the data analysis tables. Causes of medication-related adverse events for each drug in the DRUG table (drug name, causality, etc.) were classified into three categories: “suspected medicine”, “concomitant medicine”, and “interaction medicine”. We extracted only “suspected medicine” information from the DRUG table. We removed duplicated data from the DRUG and REAC tables (adverse events, outcome, etc.) [14]. Data in the DEMO table (patients’ demographic information, such as gender, age, and weight) were combined with the DRUG and REAC tables using patient identification numbers. The analytical data table was prepared by removing patients with a body mass index (BMI) of <5 or ≥100.

The flowchart for the data analysis table in this study used the JADER DRUG table with 3,514,601 records, a REAC table with 942,170 records, and a DEMO table with 595,953 records (Figure 1). We combined the three data tables and deleted 1172 ineligible records to create the data analysis table. The data analysis table contained 1,518,728 records, of which, 4597 (0.3%) were for MRONJ.

### 2.2. Patient Background and MRONJ

Most patients with MRONJ were female (3159 (71.3%); Table 1). The mean ± standard deviation for age, height, weight, and BMI were 71.4 ± 11.5 years, 154.1 ± 10.0 cm, 51.7 ± 12.1 kg, and 21.8 ± 4.2, respectively, for patients with MRONJ. The characteristics of the patients without MRONJ were 59.1 ± 21.7 years, 156.8 ± 18.9 cm, 54.3 ± 16.5 kg, and 21.9 ± 4.5, respectively. Univariate analysis identified significant differences in gender, age, height, and weight, but not in BMI. The degree of freedom for each factor was 1.

### 2.3. Medicines and MRONJ

**Figure 2:**
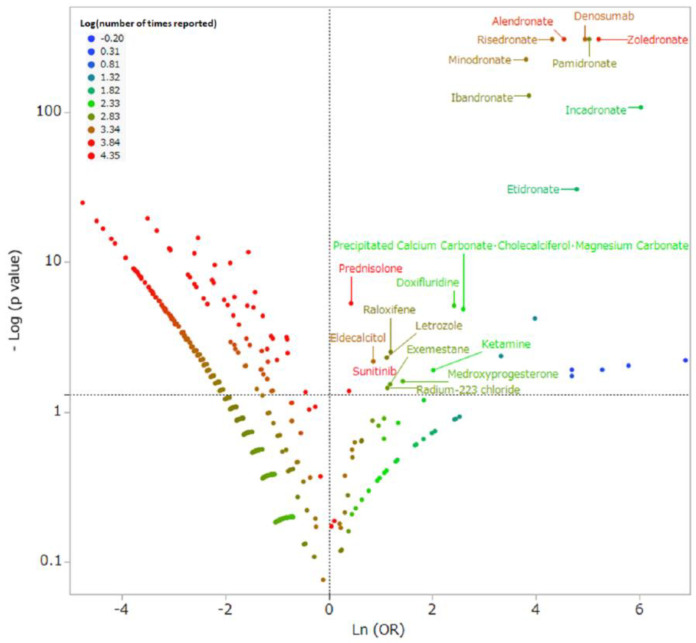
Drugs associated with medication-related osteonecrosis of the jaw. The *X*-axis shows the natural logarithms of the odds ratios (ln(OR)), and the *Y*-axis shows the common logarithm of the inverse *p*-value (−log_10_(*p*-value)) from Fisher’s exact test. The ORs were calculated using cross-tabulation (Figure 3). The dotted line on the *Y*-axis represents *p* = 0.05. Plot colors represent the number of reports of adverse events. The red–green–blue points are common logarithms of the total reported numbers (from −0.20 to 4.35). As the ORs become more positive, the tendency toward adverse events increases; decreasing *p*-values indicate a greater statistical significance. The upper-right portion of the scatter plot identifies drugs that were more likely to induce medication-related osteonecrosis of the jaw.

A scatter plot of the natural logarithms (ln(OR)) of the RORs on the *X*-axis and common logarithms of the *p*-values found using Fisher’s exact test (−log_10_(*p*-value)) on the *Y*-axis illustrates the statistical results (Figure 2). The drugs plotted in the upper-right show a significant tendency toward causing MRONJ.

The upper-right of Figure 2 shows 20 drugs that were likely to cause MRONJ (Table 2). BPs were associated with 3103 records (67.5%) of MRONJ. The most frequently reported were zoledronate in 1352 records (29.4%), alendronate in 780 records (17.0%), risedronate in 353 records (7.7%), and pamidronate in 252 records (5.5%). Denosumab was associated with 674 records (14.7%). Drugs other than BPs and denosumab were associated with 820 records (17.8%).

### 2.4. Analysis of MRONJ Using Multivariate Analysis

Multiple logistic regression identified etidronate, incadronate, pamidronate, denosumab, zoledronate, alendronate, risedronate, minodronate, ibandronate, medroxyprogesterone, exemestane, letrozole, doxyfluridine, raloxifene, prednisolone, eldecalcitol, radium chloride, sunitinib, female, elderly, and lesser height as important risk factors for MRONJ (Table 3). Precipitated calcium carbonate/cholecalciferol/magnesium carbonate and ketamine could not be included in the analysis. The local outlier factor value was *p* = 1.000 with R^2^ = 0.427.

### 2.5. Characteristics of the Side Effects Related to Osteonecrosis Using Principal Component and Cluster Analyses

The contribution ratios of the principal components were 61.7%, 16.6%, and 7.1% for the first, second, and third components, respectively. A scatterplot was created using the first two principal components.

The relationship between the side effects related to osteonecrosis and the main components was visualized with a plot, where each side effect was represented as a loading vector (Figure 4a). The *X*-axis represents the first component, and all side effect vectors showed a positive correlation. The *Y*-axis represents the second component, where atypical femoral fracture (0.717), osteomyelitis (0.665), and osteonecrosis of the jaw (0.542) showed significant positive correlations; groin pain (−0.573), bacterial osteomyelitis (−0.570), and alveolar osteitis (−0.341) showed significant negative correlations.

Most BPs and denosumab showed positive correlations with both the first and second principal components (Figure 4b). Sunitinib showed a notable negative correlation with the first principal component.

The dendrogram generated by the hierarchical cluster analysis resulted in two clusters (Figure 5). One cluster was associated with MRONJ, osteomyelitis, atypical femoral fracture, and osteonecrosis; this group showed a strong positive correlation with both the first and second principal components. The second cluster included all other side effects.

## 3. Discussion

### 3.1. Risk Factor of MRONJ

This study reports two findings regarding MRONJ. First, females and the elderly, short stature, eight types of BPs, denosumab, prednisolone, sunitinib, eldecalcitol, raloxifene, letrozole, doxifluridine, exemestane, medroxyprogesterone, and radium chloride were all factors associated with MRONJ.

The patient factors were female gender, old age, and short stature (Table 3). Vahtsevanos and colleagues reported that women and MRONJ may be related through underlying disorders, such as osteoporosis and breast cancer, in a cohort study of multiple myeloma and breast and prostate cancers [15]. Furthermore, the proportion of men with MRONJ who received BPs for osteoporosis was small [16]. In this study, 3159 (71.3%) MRONJ cases were female (Table 2). In the Japan National Survey, the average age of the patients with MRONJ was 74.6 years [17]. In this study, the mean age of patients with MRONJ was 71.4 years. No previous report indicates a correlation with lesser height; this association is new. This study suggests the possibility of screening based on patient characteristics, as do previous reports.

Drugs related to MRONJ were eight types of BPs, denosumab, prednisolone, sunitinib, eldecalcitol, raloxifene, letrozole, doxyfluridine, exemestane, radium chloride, and medroxyprogesterone (Table 3). All eight types of BPs used in Japan were correlated with MRONJ. BP has a strong affinity for bone hydroxyapatite, suppresses osteoclast activity, reduces bone resorption, and is used to treat osteoporosis and malignant tumors [18]. BPs are the most important drug associated with MRONJ, and the incidence of BPs used for malignant tumors is higher than that for treatment of osteoporosis, where it is reported to be approximately 1% [2]. On the other hand, in the Japan National Survey from 2011 to 2013, 45.3% of MRONJ cases were related to BPs used for osteoporosis [19]. In this study, zoledronic acid used for malignant tumors was most frequently reported, and alendronate, risedronate, and minodronate used for osteoporosis were frequently reported (Table 2). Thus, not only should the use of BPs for malignant tumors be particularly monitored but also its use for osteoporosis. The multiple logistic regression analysis revealed that etidronate was associated with MRONJ (Table 3). Etidronate is a non-N-containing BP and has a weaker bone resorption effect than N-containing BPs [20]. However, non-N-containing BPs have been reported to be associated with MRONJ [21]. Because this study supported previous findings, caution may be required, even in cases of non-N-containing BPs.

Denosumab showed similar odds ratios to BPs in the present study (Table 3). Denosumab is an anti-receptor activator of nuclear factor-κB ligand (RANKL) antibody and is a bone resorption inhibitor used for malignant tumors and osteoporosis [22,23]. Saad et al. reported that the incidence of MRONJ after denosumab administration for malignant bone tumors is about the same as or higher than that after BP treatment [6]. This study was consistent with the previous finding. In contrast, Baron et al. reported that denosumab had a half-life of approximately 26 days, which was shorter than that of BPs [24]. Thus, although denosumab has a short half-life, its effect on MRONJ may require the same caution as that required for BPs.

Sunitinib exhibited some influence on MRONJ. Sunitinib is an antiangiogenic agent with tyrosine kinase inhibitory action. Brunello et al. reported the exacerbation of MRONJ with sunitinib in renal cell carcinoma [25]. The current study is consistent with this previous finding. Many antiangiogenic agents are associated with MRONJ [26]. This effect of antiangiogenic agents may differ depending on their mechanism of action [7].

Several other drugs were also associated with MRONJ. Prednisolone was also correlated with MRONJ. The long-term use of corticosteroids increases the risk of developing osteonecrosis or avascular necrosis [27], and patients concomitantly administered with antiresorptive agents could have an increased risk of developing MRONJ [28]. Corticosteroids may also delay wound healing via immunosuppression, alter oral microbiota, and increase the risk of oral infection and MRONJ [6,29]. Selective estrogen receptor adjustment medications and oral contraceptives and sex hormone preparations influence the development of MRONJ. A few reports stating that such drugs are associated with MRONJ exist. Sex hormones, such as estrogen, are reported to affect bone remodeling [30] and may influence jaw bone remodeling. Radium chloride is a radiopharmaceutical that may affect MRONJ. Radium chloride suppresses osteoclasts [31]. This effect is similar to that of BP and denosumab. In contrast, radium chloride has been reported to be used for the non-surgical management of MRONJ [32]. This study was different from the previous report. This drug association has not been studied well and requires further investigation. The present comprehensive analysis suggested that many drugs could be associated with MRONJ.

Although MRONJ that is caused by antiresorptive and antiangiogenic agents is well known, it is also reported to be induced by other drugs. In this study, all drugs reported in the side effect database were comprehensively examined under the same analysis conditions. The fact that some of the bone resorption inhibitors and antiangiogenic agents were detected as notable MRONJ-inducing drugs through this analysis method ensures the credibility of the other detected drugs.

### 3.2. Relationship between MRONJ and Related Diseases

The second finding suggests that BPs and denosumab show similar osteonecrosis-related side effects and effects on osteoclasts. Furthermore, atypical femoral fractures, osteomyelitis, osteonecrosis, and MRONJ showed similar onset trends. Principal component analysis is a method for encapsulating related information into summary indices (principal components) that are more readily visualized and analyzed [33]. Each side effect and drug in this study was interpreted on the basis of principal components. The main component was interpreted using loading vectors that represent the side effect (Figure 4a). All side effect vectors were positively correlated for the first principal component. Thus, the first principal component is a comprehensive index of osteonecrosis-related side effects.

Side effect vectors were divided into those with positive and negative relationships for the second principal component. The positive side effects were atypical femoral fracture, osteomyelitis, and MRONJ. Atypical femoral fracture is reported to be affected by BP-induced osteoclast suppression [34]. Some patients with MRONJ have jaw osteomyelitis [35]. Thus, some patients with MRONJ with jaw osteomyelitis may have been reported as osteomyelitis. Conversely, the negative side effects of the second principal component were groin pain, bacterial osteomyelitis, and alveolar osteomyelitis. These side effects are considered to show little effect on osteoclasts. Thus, the second principal component is a comprehensive index related to osteoclasts.

Drugs were also interpreted using a score plot (Figure 4b). Most BPs showed a positive correlation with both the first and second principal components. The principal component analysis suggested that osteoclasts were involved in the side effects of BPs, such as MRONJ and atypical femoral fractures. On the other hand, no difference was observed in the osteonecrosis-related side effects between the BPs used for malignant tumors and the BPs used for osteoporosis in the principal component analysis of this study. Denosumab, an anti-RANKL antibody, showed a positive correlation with both the first and second principal components. Thus, the osteonecrosis-related side effects of denosumab involved osteoclasts. Furthermore, denosumab plotted near the BPs. The effect of denosumab on osteoclasts was similar to that of BPs. Conversely, the antiangiogenic agent sunitinib was plotted separately from BPs and denosumab. The effect of sunitinib on osteonecrosis may occur via a different mechanism than that of osteoclast-mediated BPs and denosumab.

The cluster analysis in this study showed similar features for atypical femoral fractures, osteomyelitis, osteonecrosis, and MRONJ (Figure 5). Cluster analysis classifies data into similar groups (clusters) [36]. Thirteen side effects were divided into two clusters in this study, and the cluster characteristics were evaluated. First, one cluster was MRONJ, osteomyelitis, atypical femoral fracture, and osteonecrosis. This cluster showed a strong positive correlation with the first and second principal components and was strongly associated with the osteonecrosis activity of osteoclasts. Furthermore, this cluster was also associated with BP and denosumab. Conversely, the second cluster encompassed all other side effects. This cluster was characterized by many side effects that had little association with osteoclasts. Osteonecrosis of the external auditory meatus was not associated with MRONJ.

Atypical femoral fractures had similar characteristics to MRONJ. These fractures are atraumatic fractures of the subtrochanteric and proximal femoral shaft [37]. Odvina et al. reported that atypical femoral fractures are primarily caused by severely suppressed bone turnover due to long-term suppression by BPs; chronic administration adversely affects material properties and the strength of bone [34]. The mechanism of an atypical femoral fracture is, therefore, due to the inhibition of osteoclast activity and is similar to MRONJ [38,39]. The features of atypical femoral fractures in this study support existing findings. Additionally, denosumab showed similar effects to BPs. Therefore, high-risk patients with MRONJ should be monitored for atypical femoral fractures.

### 3.3. Limitations

This study has three limitations [40,41]. First, the database was based on spontaneous reports, and cases were limited by the recognition of side effects. The total number of patients was not known, and a true assessment of side effects was not possible. Second, JADER data have blank cells, and some reports have incorrect characters and numbers. Therefore, this study revised the side effects and drug names as much as possible and evaluated the patient background using the BMI. Third, the cause of side effects is difficult to determine when multiple drugs are administered. Fatal side effects are verified by the PMDA, but other side effects are entered at the discretion of the reporter. Conversely, JADER is the largest database of reported adverse drug reactions in Japan. Side effect information obtained from JADER reflects unique pharmacological and pharmacokinetic characteristics and also prescription and usage status. JADER is used in various research areas and remains an excellent tool for inductively evaluating the side effects of drugs.

## 4. Materials and Methods

### 4.1. JADER and Production of the Data Analysis Table

JADER is a voluntary reporting database that is made publicly available by the Pharmaceuticals and Medical Devices Agency (PMDA). In Japan, JADER is the largest database that can be used to investigate side effects. JADER was downloaded from the PMDA website [42]. JADER data used in the study included 595,953 adverse drug reactions reported from 1 April 2004 to 30 September 2019 (Figure 1). Case reports are presented in JADER in four tables: DRUG (drug name, causality, etc.), REAC (adverse events, outcome, etc.), DEMO (patients’ demographic information, such as gender, age, and weight), and HIST (medical history, primary illness, etc.). Three tables were used, namely, the DRUG, REAC, and DEMO tables. Drugs registered in the DRUG table are categorized as suspected drugs, concomitant drugs, and interactions, depending on their involvement in side effects. Only data for suspected drugs were extracted from the DRUG table. Adverse events in the REAC table are recorded by preferred terms from the International Council for Harmonisation of Technical Requirements for Pharmaceuticals for Human Use of Pharmaceutical Terms (Medical Dictionary for Regulatory Activities Japanese version 18.0 (MedDRA/J v18.0)) [43]. We removed duplicate data from the DRUG and REAC tables using methods described by Hirooka et al. [14,44]. Patients with the same identification number were merged in the tables to eliminate duplicating information. The three tables (DRUG, REAC, and DEMO) were merged using the identification number, and the joined data were cleaned using the body mass index (BMI). The BMI was calculated from the combined data tables and outliers with a BMI < 5 (*n* = 8) or BMI ≥ 100 (*n* = 1164) were treated as abnormal values and removed from the analytical data table (Figure 1). In this study, cases reported as osteonecrosis of the jaw were defined as MRONJ.

### 4.2. Relationship between Patient Information and MRONJ

The data analysis table was divided into MRONJ and non-MRONJ groups for comparative studies. We treated age, height, and weight data as absolute numbers, and calculated *p*-values using Wilcoxon’s rank-sum test. A weight of over 60 kg was assumed to be 65 kg, and a weight under 10 kg was assumed to be 5 kg. For sex, *p*-values were calculated using Fisher’s exact test. Patient factors were analyzed only with data sets that did not include missing values.

### 4.3. Relationship between Medicines and MRONJ

**Figure 3:**
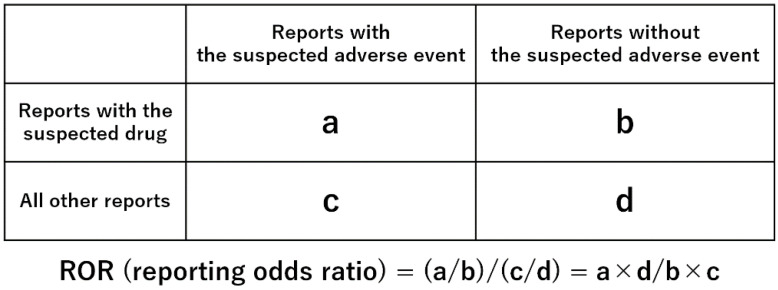
Cross-tabulation and calculation formula of the reported odds ratios (RORs) for adverse events. The cross-tabulation is structured with reports for the suspected drug, all other reports, reports with adverse events, and reports without adverse event (a–d indicate the numbers of reports). The RORs are provided as odds ratios (ORs).

**Figure 4:**
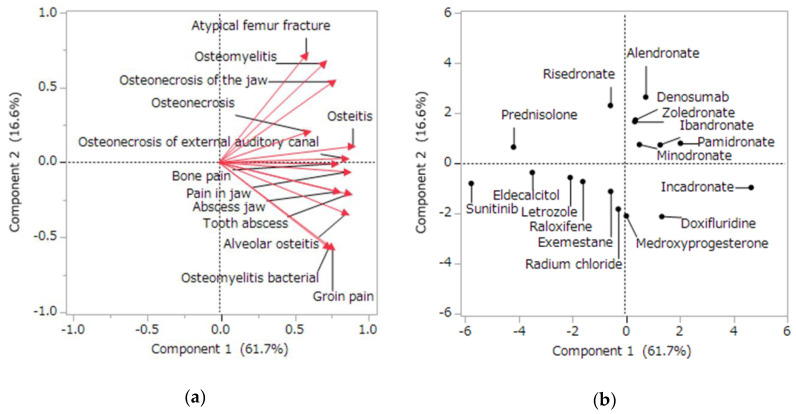
Relationships between the osteonecrosis-related side effects and drugs using principal component analysis. Loading vectors represent the relationship between the side effects and principal components (**a**). Each loading vector indicates a side effect. The score plot shows the relationships between the drugs and principal components (**b**). Each dot indicates a drug.

**Figure 5:**
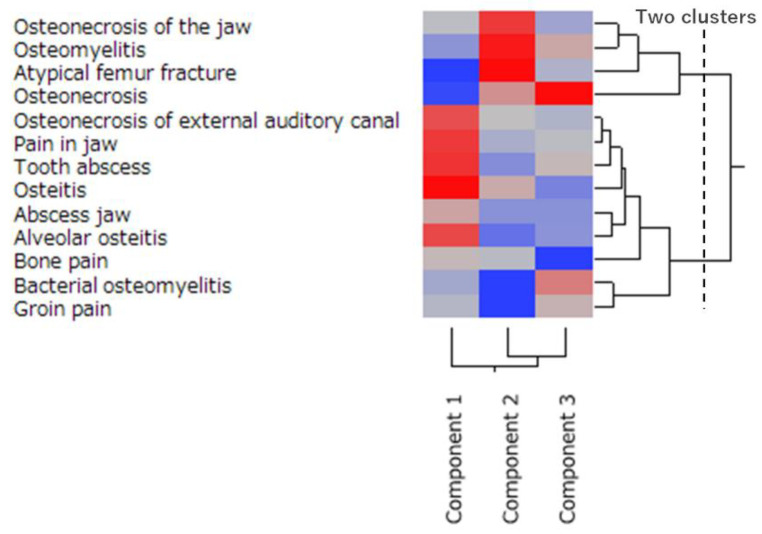
Classification of the osteonecrosis-related side effects using hierarchical cluster analysis. The dendrogram shows the relationships between 13 types of osteonecrosis-related side effects. The dotted line shows the separation of the clusters. The color map shows the load value of the principal components in red–gray–blue (high to low load values).

We analyzed 4327 drugs reported as suspected drugs under common names. We assessed the risks of MRONJ for each drug using reported odds ratios (RORs) and Fisher’s exact test. Initially, we created a 2 × 2 contingency table of drugs and side effects for each drug (Figure 3). A 2 × 2 contingency table cannot be calculated with 0 cells, and the estimation is unstable when the cell frequency is small. Therefore, 0.5 was added to all cells as a correction (Haldane Anscombe half correction) [45,46]. In this study, MRONJ-related drugs were defined as those with ROR ≥ 1 and a Fisher’s exact test *p*-value < 0.05 [47]. Subsequently, a scatterplot consisting of ROR and *p*-values was created for a visual interpretation of the drug effects. This scatter plot was created by converting ROR to the natural logarithms of the odds ratio (ln(OR)) and converting the *p*-value of the Fisher’s exact test to common logarithms (−log_10_(*p*-value)). The scatter plot corresponds to volcano plots frequently used to understand gene expression trends in bioinformatics [48,49].

### 4.4. Multivariate Analysis

We performed multiple logistic regression to identify the factors related to MRONJ. The independent variable was the presence or absence of MRONJ. The dependent variables were the patient background and drugs that were significantly correlated in the previous univariate analysis [50]. The multiple logistic regression analysis used 752,993 records, excluding the reports with missing values out of the 1,517,553 records in the data analysis table.

### 4.5. Analysis of the Side Effects Related to Osteonecrosis Using Principal Component and Cluster Analyses

We used principal component and cluster analyses to assess the osteonecrosis reported in JADER. Initially, we examined the onset of the osteonecrosis-related side effects of 18 drugs associated with MRONJ using multiple logistic regression analysis. The side effects reported to JADER are grouped by standardized MedDRA queries (SMQ) in terms of the medical condition and area [51]. Osteonecrosis (SMQ; 2000018), including MRONJ, was used. We examined 13 osteonecrosis-related side effects with a reported number of ≥20: osteonecrosis of the jaw (*n* = 4597), osteonecrosis (*n* = 1476), osteomyelitis (*n* = 1486), atypical femoral fracture (*n* = 1347), bone pain (*n* = 174), pain in the jaw (*n* = 116), jaw abscess (*n* = 79), osteitis (*n* = 76), tooth abscess (*n* = 56), bacterial osteomyelitis (*n* = 34), osteonecrosis of the external auditory canal (*n* = 28), groin pain (*n* = 26), and alveolar osteitis (*n* = 21). Subsequently, we calculated the ROR from 2 × 2 contingency tables for 13 side effects and 18 drugs. The RORs were converted to natural logarithms and used in the principal component analysis with correlation matrices [33,52]. The first and second principal components were used to interpret the characteristics of the drugs and their side effects. Furthermore, a hierarchical cluster analysis was used to objectively classify the side effects. This analysis used the Ward method based on Euclidean distance with loads (cumulative contribution rate: 85.5%) from the first, second, and third principal components [36,53]. The hierarchical cluster analysis established two clusters.

### 4.6. Statistical Analysis

All analyses were performed using JMP Pro13.2.0 (SAS Institute Inc., Cary, NC, USA), and the level for statistical significance was set to 0.05.

## 5. Conclusions

MRONJ is a rare side effect, but we were able to analyze a large dataset of MRONJ cases. Two findings were revealed. First, MRONJ appeared to be related to 18 drugs and some patient characteristics. Second, several drugs were associated with atypical femoral fractures, as well as MRONJ. We expect our results to contribute to the appropriate management of drug side effects by healthcare professionals.

## Figures and Tables

**Table 1:** Patient backgrounds.

Patients	MRONJ (4597)	Non-MRONJ (1,517,553)	*p*-Value
Gender ^#^ (male/female)	1270/3159 (4429)	754,933/711,784 (1,466,717)	<0.0001 ^##^
Age ^†^	71.4 ± 11.5 (4230)	59.1 ± 21.7 (1,416,925)	<0.0001 **
Height (cm) ^†^	154.1 ± 10.0 (1477)	156.8 ± 18.9 (650,399)	<0.0001 **
Weight (kg) ^†^	51.7 ± 12.1 (1567)	54.3 ± 16.5 (761,613)	<0.0001 **
BMI ^†^	21.8 ± 4.2 (1451)	21.9 ± 4.5 (628,976)	0.4802

MRONJ, medication-related osteonecrosis of the jaw; BMI, body mass index. Each item included some missing values. Analyses were performed using data after eliminating these records. The numbers in parentheses are the numbers of causes used in the analyses. ^#^: Fisher’s exact test; ^†^: Wilcoxon signed-rank test; ^##,^** *p* < 0.001.

**Table 2:** Results of the Fisher’s exact test based on the presence or absence of osteonecrosis of the jaw (*N* = 1,512,959).

Medicine	Drug Class	Reporting Times	Reporting Ratio (%)	Odds Ratio	95% Confidence Interval	*p*-Value
Zoledronate	BP	1352	29.41	184.40	171.63–198.12	<0.0001 **
Alendronate	BP	780	16.97	94.06	86.46–102.33	<0.0001 **
Denosumab	RANKL inhibitor	674	14.66	141.22	128.60–155.08	<0.0001 **
Risedronate	BP	353	7.68	74.90	66.52–84.33	<0.0001 **
Pamidronate	BP	252	5.48	153.50	131.98–178.53	<0.0001 **
Minodronate	BP	188	4.09	45.27	38.78–52.84	<0.0001 **
Prednisolone	Corticosteroid	137	2.98	1.53	1.29–1.81	<0.0001 **
Ibandronate	BP	105	2.28	47.83	38.92–58.77	<0.0001 **
Incadronate	BP	54	1.17	417.19	279.77–622.09	<0.0001 **
Sunitinib	Tyrosine kinase inhibitor	32	0.70	1.48	1.04–2.09	0.0416 *
Etidronate	BP	19	0.41	120.44	71.66–202.44	<0.0001 **
Eldecalcitol	Vitamin D	14	0.30	2.36	1.40–3.95	0.0067 *
Raloxifene	SERM	9	0.20	3.29	1.74–6.24	0.0031 *
Letrozole	Aromatase inhibitor	9	0.20	3.05	1.61–5.79	0.0049 *
Doxifluridine	Pyrimidine fluoride drug	7	0.15	11.26	5.44–23.33	<0.0001**
Precipitated Calcium Carbonate･ Cholecalciferol･ Magnesium Carbonate	Calcium･ vitamin D･ magnesium	6	0.13	13.43	6.13–29.43	<0.0001 **
Exemestane	Aromatase inhibitor	5	0.11	3.26	1.41–7.56	0.0294 *
Radium chloride	Radiopharmaceutical	5	0.11	3.09	1.33–7.15	0.0357 *
Medroxyprogesterone	Progestogen	4	0.09	4.17	1.64–10.57	0.0248 *
Ketamine	Anesthetic	3	0.07	7.51	2.60–21.67	0.0124 *

BP, bisphosphonate; RANKL, human monoclonal antibody to the receptor activator of nuclear factor-κB ligand; SERM, selective estrogen receptor modulator. “Reporting Times” shows the number of cases that reported a medicine suspected as a cause of osteonecrosis of the jaw. *: *p* < 0.05, **: *p* < 0.0001.

**Table 3:** Multiple logistic regression analysis using medication and patient variables (*N* = 752,993).

Risk Factor	Odds Ratio	95% Confidence Interval	*p*-Value
Etidronate	1609.16	680.33–3806.08	<0.0001 **
Incadronate	1258.09	613.01–2582.01	<0.0001 **
Pamidronate	963.56	713.45–1301.35	<0.0001 **
Denosumab	775.18	640.35–938.41	<0.0001 **
Zoledronate	733.31	610.87–880.28	<0.0001 **
Alendronate	404.38	328.08–498.42	<0.0001 **
Risedronate	288.97	218.07–382.91	<0.0001 **
Minodronate	264.79	191.03–367.03	<0.0001 **
Ibandronate	216.06	136.45–342.12	<0.0001 **
Medroxyprogesterone	42.10	10.33–171.72	<0.0001 **
Exemestane	37.30	11.80–117.91	<0.0001 **
Letrozole	34.65	12.76–94.08	<0.0001 **
Doxifluridine	33.07	4.58–238.87	0.0005 *
Raloxifene	19.91	7.35–53.97	<0.0001 **
Prednisolone	19.75	14.98–26.05	<0.0001 **
Eldecalcitol	16.11	7.10–36.58	<0.0001 **
Radium chloride	13.13	1.83–94.25	0.0104 *
Sunitinib	9.76	5.45–17.50	<0.0001 **
Female	1.28	1.10–1.48	0.0012 *
Unit Odds Ratio			
Risk Factor	Odds Ratio	95% Confidence Interval	*p*-Value
Age	1.02	1.01–1.02	<0.0001 **
Height	0.99	0.985–0.998	0.0111 *
Weight	1.00	0.997–1.008	0.4120
Range Odds Ratio			
Risk Factor	Odds Ratio	95% Confidence Interval	*p*-Value
Age	6.24	4.08–9.55	<0.0001 **
Height	0.23	0.07–0.71	0.0111 *
Weight	1.61	0.52–5.03	0.4120

Each item included some missing values. Analyses were performed using data after eliminating these records. *: *p* < 0.05, **: *p* < 0.0001.
